# Labetalol Prevents Intestinal Dysfunction Induced by Traumatic Brain Injury

**DOI:** 10.1371/journal.pone.0133215

**Published:** 2015-07-17

**Authors:** Yuhuang Lang, Fengming Fu, Dalong Sun, Chenhui Xi, Fengyuan Chen

**Affiliations:** 1 Department of Emergency, Shanghai Fifth Hospital affiliated Fudan University, Shanghai, China; 2 Intensive Care Unit, Fudan University Shanghai Cancer Center, Shanghai, China; 3 Department of Gastroenterology, Shanghai Fifth Hospital affiliated Fudan University, Shanghai, China; University of California, Los Angeles, UNITED STATES

## Abstract

**Background:**

Beta-adrenergic blockade has been hypothesized to have a protective effect on intestinal dysfunction and increased intestinal permeability associated with the epinephrine surge after traumatic brain injury (TBI).

**Methods:**

Wister rats were subjected to either a weight drop TBI, and intraperitoneally injected or not with labetalol, or a sham procedure (18 rats per group). After 3, 6, or 12h (6 rats per subgroup), intestinal permeability to 4.4 kDa FITC-Dextran and plasma epinephrine levels were measured as was intestinal tight junction protein ZO-1 expression at 12h. Terminal ileum was harvested to measure levels of intestinal tumor necrosis factor (TNF)-α and to evaluate histopathology.

**Results:**

In TBI group vs. sham group, intestinal permeability (P<0.01) was significantly higher at all time-points, and intestinal ZO-1 expression was lower at 12h. In TBI with vs. without labetalol group, 1) intestinal permeability was significantly lower at 6 and 12h (94.31±7.64 vs. 102.16±6.40 μg/mL; 110.21±7.52 vs. 118.95±7.11 μg/mL, respectively); 2) levels of plasma epinephrine and intestinal TNF-α were significantly lower at 3, 6 and 12h; and 3) intestinal ZO-1 expression was higher at 3, 6 and 12h (p=0.018). Histopathological evaluation showed that labetalol use preserved intestinal architecture throughout.

**Conclusion:**

In a rat model of TBI, labetalol reduced TBI-induced sympathetic hyperactivity, and prevented histopathological intestinal injury accompanied by changes in gut permeability and gut TNF-α expression.

## Introduction

Traumatic brain injury (TBI) is the leading cause of death and disability in severe trauma and is drawing more and more attention. TBI can cause significant gastrointestinal alterations and impairment, including Cushing’s ulceration, inflammation, loss of intestinal tight junction proteins and increased gut permeability [1, 2]. Increased intestinal permeability leads to bacterial translocation which can in turn lead to sepsis and multiple organ failure. Intestinal mucosa structural changes, barrier dysfunction and increased intestinal permeability leading to bacterial translocation, sepsis and multiple organ failure might present as early as 3–6 hours after traumatic brain injury (TBI) [1–4]. The proposed mechanisms include increased adrenergic tone, reduced mucosal blood flow, decreased bowel movements, surge of intestinal TNF-α, and/or loss of intestinal tight junction proteins [1, 5–7].

Decreasing adrenergic tone with beta-blockers might improve outcome after TBI [8].

Several recent studies have showed at beta-blocker exposure was associated with a significant reduction in mortality in patients with severe TBI [8, 9]. Adrenergic receptor blockade can block the activation of the hypothalamus—pituitary—adrenal axis (HPA) mediated by the locus coeruleus/norepinephrine/sympathetic nervous system, thus interfering with the positive feedback path of stress response, and weakening the hyperfunction of the HPA axis under continuous stress state. Labetalol is a selective α1- and nonselective β1- and β2-adrenergic antagonist that is widely used in the treatment of hypertension [10]. Labetalol is classified among lipophilic beta-blockers, its plasma half-life is 3–6 h [11], and it is highly resorbed in the gastrointestinal tract, and metabolized by the liver into glucuronide derivates with an extensive first-pass effect. Labetalol also can slow sinus rhythm and reduce peripheral vascular resistance [12]. In light of the hyperadrenergic state that has been described after severe head injury, we hypothesized that labetalol would preserve intestinal homeostasis by preventing increased intestinal permeability after TBI.

## Materials and Methods

### Animals and TBI Model

The study protocol was approved by the Fudan University, Ministry of Science Animal Ethics Committee. Adult male Wister rats weighing 200 to 250g were purchased from the Animal Center of the Chinese Academy of Sciences, Shanghai, China. Rats were housed at 25°C with 12-hour light/dark cycles and free access to food and water.

The rats were randomly divided into three groups (18 rats each) [13, 14] including Sham group (right parietal bone window alone, without brain injury), TBI group, and TBI+labetalol group (TBI + labetalol 30 mg/kg i.p.) [15]. Labetalol (Shanghai, China) was prepared as a 0.5%working solution by dissolving 0.1 g in 20 ml of sterile saline. Working solutions were stored sterile and away from light at 25°C. Each group was further divided into three subgroups (6 rats each) for assessment at 3, 6, and 12h post injury, respectively. The weight drop TBI model described by Feeney [16] was used to create a contusion injury of the right parietal cortex. We used an improved method of chloral hydrate anesthesia in rat by intermittent intraperitoneal injection in experimental animals [17–19]. Animals received four dosages of chloral hydrate (100 mg/kg) by intraperitoneal injection at three minute intervals [19]. Following intraperitoneal anesthesia with chloral hydrate, the animal’s head was shaved with an electric clipper, and fixed in a stereotactic device. Under strict asepsis, the scalp was opened and a right parietal bone window 5 mm in diameter was created with a dental drill just behind the cranial coronal suture and next to the midline. Following the removal of a small bone flap, a circular footplate was made to rest on the surface of the dura, which remained intact. A 25cm tube guided a falling weight onto the footplate resting on the dura with forces of 1000g/cm applied (40g weight after dropping 25cm). After impact, the bone window was closed using a small bone flap and incision was closed with 3–0 silk suture in all animals and the procedure needed about 5 min. Sham group animals underwent anesthesia, scalp incision and bone window creation, without brain injury. Animals that had undergone brain injury were given intraperitoneal labetalol or normal saline after the bone window was closed, and sham procedure animals received saline. At 3, 6, or 12h after TBI±labetalol or sham procedure, animals underwent an in vivo intestinal permeability assay, followed 30 minutes later by blood collection by cardiac puncture to measure FITC-Dextran concentration and epinephrine levels, and removal of terminal ileum, which was either snap frozen for protein extraction or stored in 4% formalin for histological evaluation.

### Labetalol intervention

5 min after TBI or sham operation, respectively, the TBI+labetalol group was intraperitoneally injected with labetalol (30mg/Kg) and the sham group with an equal volume of normal saline.

### In Vivo Intestinal Permeability Assay

Animals underwent an in vivo intestinal permeability assay as described by Chen et al. [20]. At 3, 6, or 12h after TBI±labetalol or sham procedure, animals were once again anesthetized with10% chloral hydrate (300mg/kg body weight) by intraperitoneal injection. A midline incision laparotomy was performed, and a 20-cm segment of the jejunum was dissected beginning 5 cm distal to the ligament of Treitz with well-protected superior mesenteric vessels. The bilateral ends of the isolated jejunum were tied with silk to prevent FITC-Dextran leakage. Previously prepared FITC-Dextran (25 mg of 4.4-kDa FITC-Dextran in 1 mL phosphate-buffered saline) was injected into the lumen of the isolated jejunum. The isolated jejunum was returned into the abdominal cavity and the abdominal wall was closed using 3–0 silk suture. Thirty minutes after FITC-Dextran injection, blood was collected by cardiac puncture and blood samples were placed into heparinized Eppendorf tubes and centrifuged at 10,000g for 10 min. Plasma was removed and subsequently assayed using a Hitachi Fluorescence spectrophotometer-F-7000 (Hitachi, Japan) to determine the concentration of FITC-Dextran. A standard curve for the assay was obtained through serial dilution of FITC-Dextran in rat serum.

### Levels of Plasma epinephrine

The epinephrine plasma levels were measured using a commercially available ELISA kit (San Diego, CA, USA) with minimum limit of epinephrine detection of 2.8 pg/mL.

### Levels of Intestinal TNF-α

The TNF-α contents in the terminal ileum tissue were expressed as nanograms of TNF-α per gram of tissue protein. At 3, 6, or 12h after TBI±labetalol or sham procedure, the levels of intestinal tumor necrosis factor-α (TNF-α) in the tissue supernatant fluids were measured using an ELISA kit (San Diego, CA,USA) specific for rat TNF-α[21,22]. The minimum limit of TNF-α detection for this assay was 15 pg/mL. The terminal ileum tissue (100 mg per rat) was transferred into a 5 mL tube, and 1mL tissue lysis buffer (Beyotime, Shanghai, China) supplemented with protease inhibitor cocktail (Roche, 04693132001) and phosphatase inhibitor cocktail (Sigma-Aldrich, Germany) was added. The tissue was homogenized on ice using a tissue homogenizer (Pro Scientific, USA); the tissue homogenate was transferred into a Dounce tissue grinder and further processed. The homogenate then was transferred into 1.5 mL tubes and centrifuged for 15 min at 16000g at 4°C; the supernatant fluid was removed and its protein concentration was determined (Pierce Biotechnology, Rockford, USA). The samples were subsequently diluted with deionized water to achieve a concentration of 4 mg protein in 1 mL total volume.

### Western blot analysis

At 12h after TBI±Labetalol or sham procedure, protein samples of distal ileum were separated by SDS-PAGE and transferred onto PVDF membrane (0.2μm, Millipore,Massachusetts, USA). The membrane was blocked for 90 min in TTBS (0.1% (v/v) Tween 20,150 mM NaCl, and 50 mM Tris—HCl, pH 7.5) containing 5% nonfat milk and was then incubated overnight at 4°C with primary antibodies specific to ZO-1 (Santa Cruz, CA, USA), followed by incubation with the appropriate secondary antibodies(Jackson Immuno Research, PA, USA) for 90 min. The dilutions of the primary antibodies were as follows: ZO-1, 1:100; and anti-β-actin (Sigma, MO, USA), 1:4,000. The dilution of the secondary antibody was 1:8,000. After thorough washing, immunoreactive bands were detected using enhanced chemiluminescence (ECL Plus, NJ, USA), according to the manufacturer’s instructions. Relative band density was calculated by dividing the pixel density of each sample by the mean pixel density of sham samples [23, 24].

### Real-time quantitative PCR

Total RNA was isolated from tissues or cells using TRIzol Reagent (Invitrogen, Shanghai, China) and purified using the RNeasy Total RNA Isolation Kit (Qiagen, Hilden, Germany). Real-time quantitative PCR was performed using the Real-Time PCR System (Biosystems, CA, USA) and the Perfect Real Time Kit (SYBR, Dalian, China). For the rat gene expression, the following SYBR Green real-time PCR primers were used: ZO-1 forward, 5′-AGTTCTGCCCTCAGCTACCA-3′ and reverse, 5′-GCTTAAAGCTGGCAGTGTC-3′; and β-actin forward, 5-CCTAGACTTCGAGCAAGAGA-3′ and reverse 5′-AGAGGTCTTTACGGATGTCA-3′.

### Histological Evaluation

After heart puncture blood collection, segments of distal ileum (obtained 5 cm away from the ileocecal junction) were dehydrated, embedded in paraffin and stained with hematoxylin and eosin (H&E) [25, 26]. Using light microscopy (Olympus, Japan), a pathologist blinded to the groups quantified pathological changes of the intestinal mucosal using mucosal damage index and Chiu's score [27] as follows: 0, Villous structure of the normal mucosa; I: intestinal mucosal villi over widened subepithelial space; II: villi with subcutaneous gap, further expansion of the villus tip epithelial elevation, and lamina propria stripped; III: villous both sides of the epithelium into a block off; IV: epithelium completely off, with lamina propria as only remaining structure; and V: mucosal lamina propria off, bleeding and ulcers.

### Statistical Analysis

SPSS software 17.0 (Chicago, USA) was used for statistical analysis. All data are expressed as mean±SD and were compared using one-way ANOVA analysis. Statistical significance was accepted at *p*<0.05.

## Results

### Intestinal Permeability

**Fig 1:**
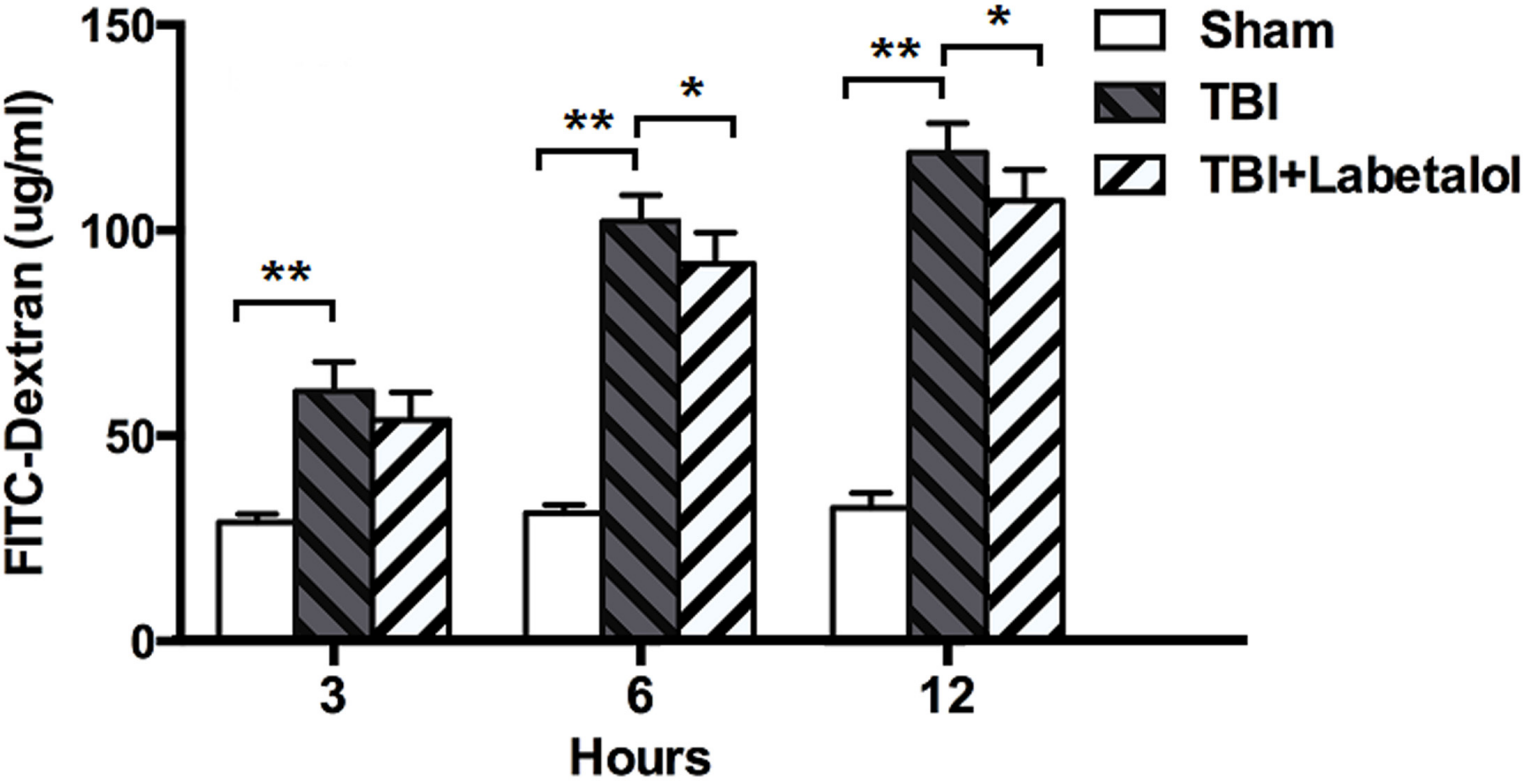
FITC-Dextran levels classified in sham, TBI and TBI+labetalol groups at 3, 6 and 12h. Intestinal permeability was higher at all time-points in TBI vs. sham group (**p <0.01), and lower in TBI with vs. without labetalol at 6 and 12h (94.31±7.64 vs. 102.16±6.40 μg/mL; and 110.21±7.52 vs. 118.95±7.11 μg/mL, respectively; *p<0.05).

In vivo intestinal permeability as determined by spectrophotometric measurement of plasma 4.4 kDa FITC-Dextran, was higher in TBI vs. sham group at all time-points (3h: 60.82±7.13 vs. 28.94±2.05 μg/mL; 6h: 102.16±6.40 vs. 31.21±1.92 μg/mL; and 12h: 118.95±7.11 vs. 32.39±3.67 μg/mL; *p*<0.01), and lower at 6 and 12h in TBI with vs. without labetalol groups (94.31±7.64 vs. 102.16±6.40 μg/mL; and 110.21±7.52 vs. 118.95±7.11 μg/mL, respectively; *p*<0.05) (Fig 1).

### Plasma epinephrine levels

**Fig 2:**
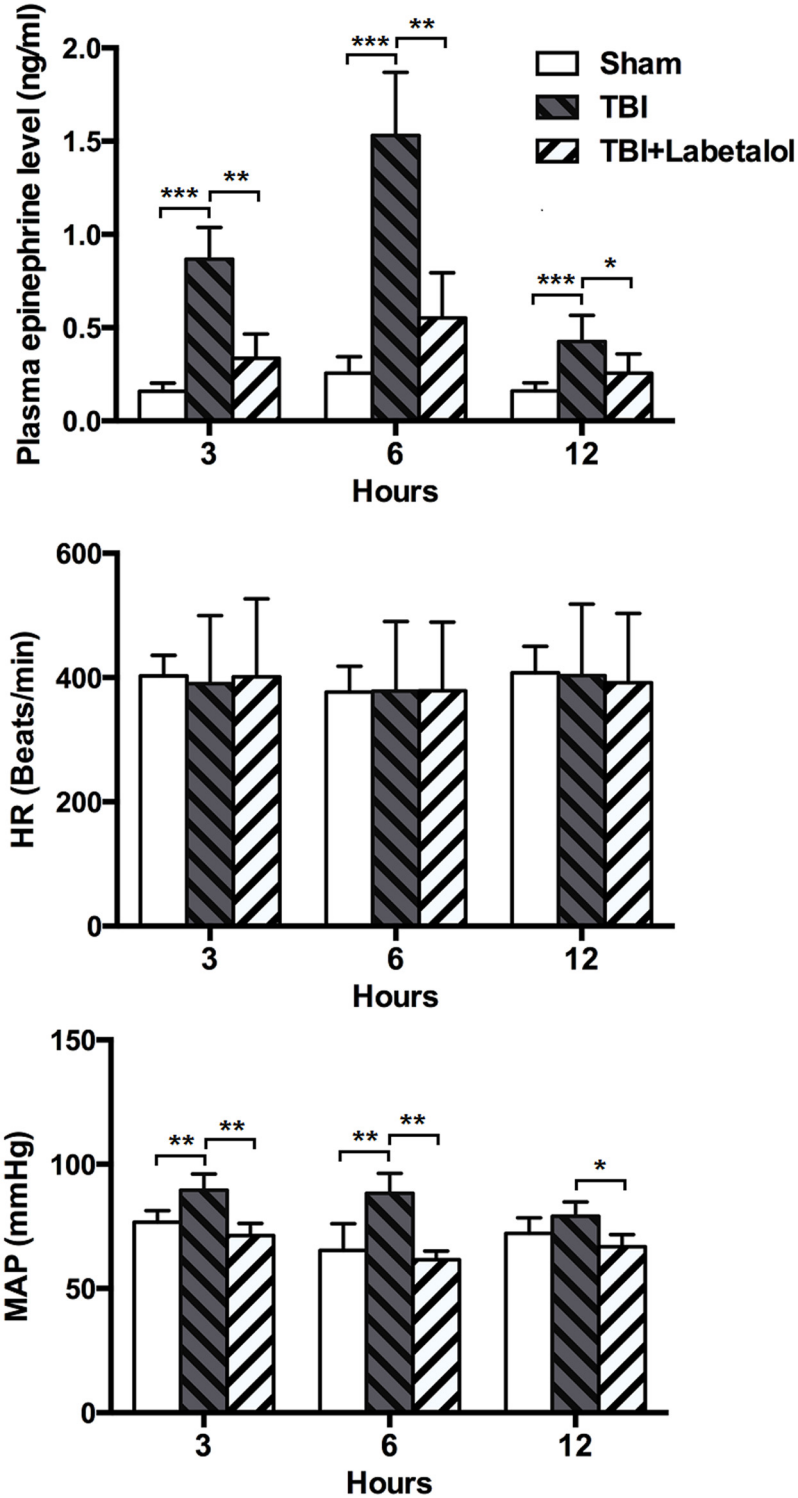
Plasma epinephrine levels in sham, TBI and TBI+labetalol groups at 3, 6 and 12h. Plasma epinephrine levels were significantly higher in TBI vs. sham group at all time-points (***p<0.001), and lower for TBI with vs. without i.p. labetalol at 3 and 6h (0.34±0.13 vs. 0.87±0.17ng/mL; and 0.55±0.24 vs. 1.53±0.34 ng/mL, respectively, **p <0.01), and 12h (0.26±0.10 vs. 0.43±0.14 ng/mL; *p<0.05).

At all time-points, plasma epinephrine levels were higher in TBI vs. sham group (3h: 0.87±0.17 vs. 0.16±0.04 ng/mL; 6h: 1.53±0.34 vs. 0.26±0.08ng/mL; and 12h: 0.43±0.14 vs. 0.16±0.04 ng/mL, all *p<0*.*01*), and lower in TBI with vs. without labetalol (3h: 0.34±0.13 vs. 0.87±0.17 ng/mL; 6h: 0.55±0.24 vs. 1.53±0.34ng/mL; and 12h: 0.26±0.10 vs. 0.43±0.14 ng/mL; *p*<0.01, *p*<0.05)(Fig 2).

### Intestinal TNF-α Levels

**Fig 3:**
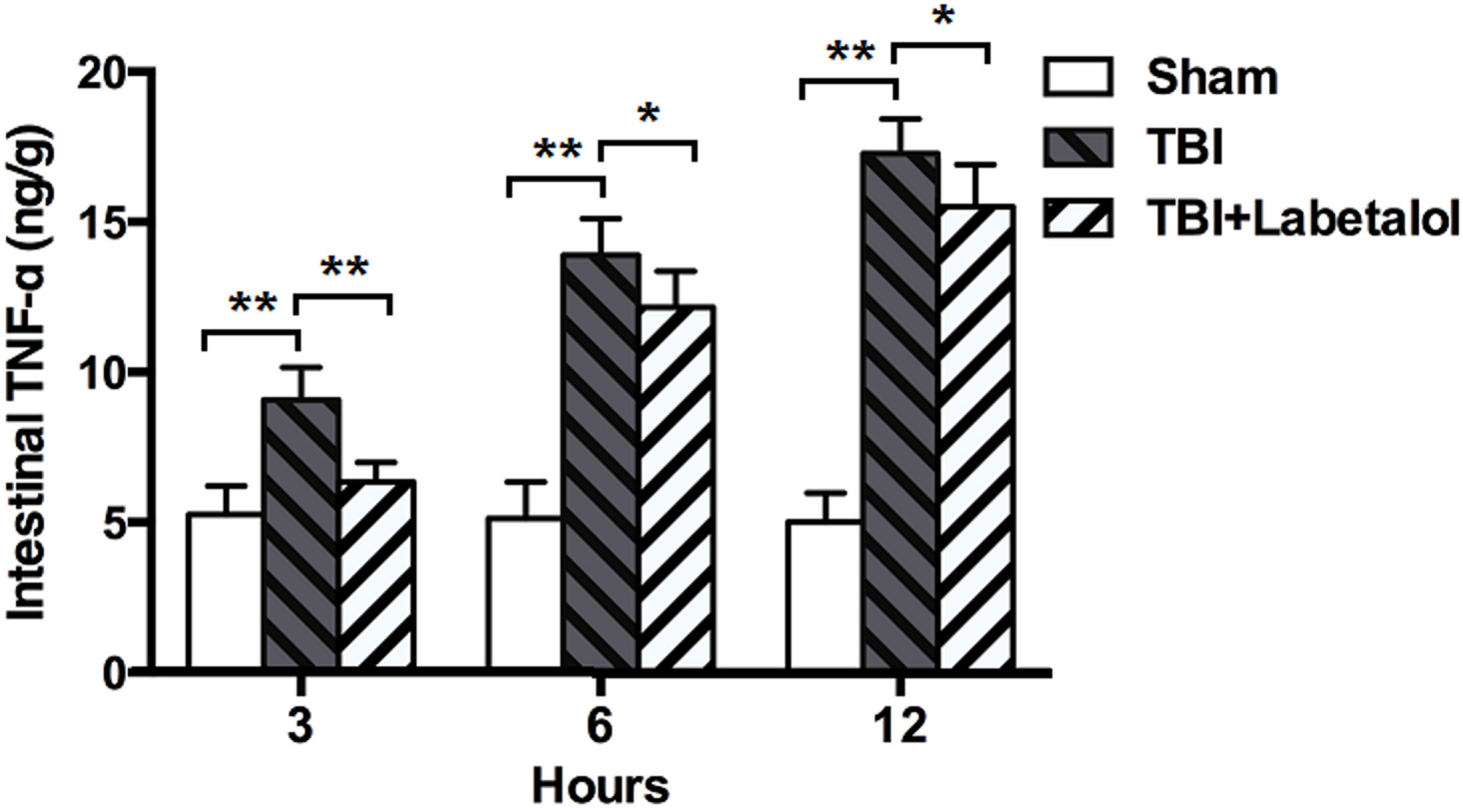
Intestinal TNF-α levels classified in sham, TBI and TBI+labetalol groups at 3, 6 and 12h. At all time-points, intestinal TNF-α was higher in TBI groups vs. sham group, and lower in TBI with vs. without labetalol (3h: 6.34±0.65 vs. 9.07±1.09 ng/g; 6 h: 12.15±1.21 vs. 13.90±1.22 ng/g; and 12 h: 15.70±1.41 vs. 17.28±1.15 ng/g; **p<0.001, *p<0.05)

At all time-points, TNF-α levels were higher in the intestinal homogenates of TBI vs. sham group (3h: 9.07±1.09 vs. 5.27±0.95 ng/g; 6h: 13.89±1.21 vs. 5.12±1.22 ng/g; and 12h: 17.28±1.15vs. 5.01±0.96 ng/g; *p*<0.01); and lower in TBI with vs. without labetalol (3h: 6.34±0.65 vs. 9.07±1.09 ng/g; 6h: 12.15±1.21 vs. 13.90±1.22 ng/g; and 12h: 15.70±1.41 vs. 17.28±1.15 ng/g; *p*<0.001, *p*<0.05) (Fig 3)

### ZO-1 expression

**Fig 4:**
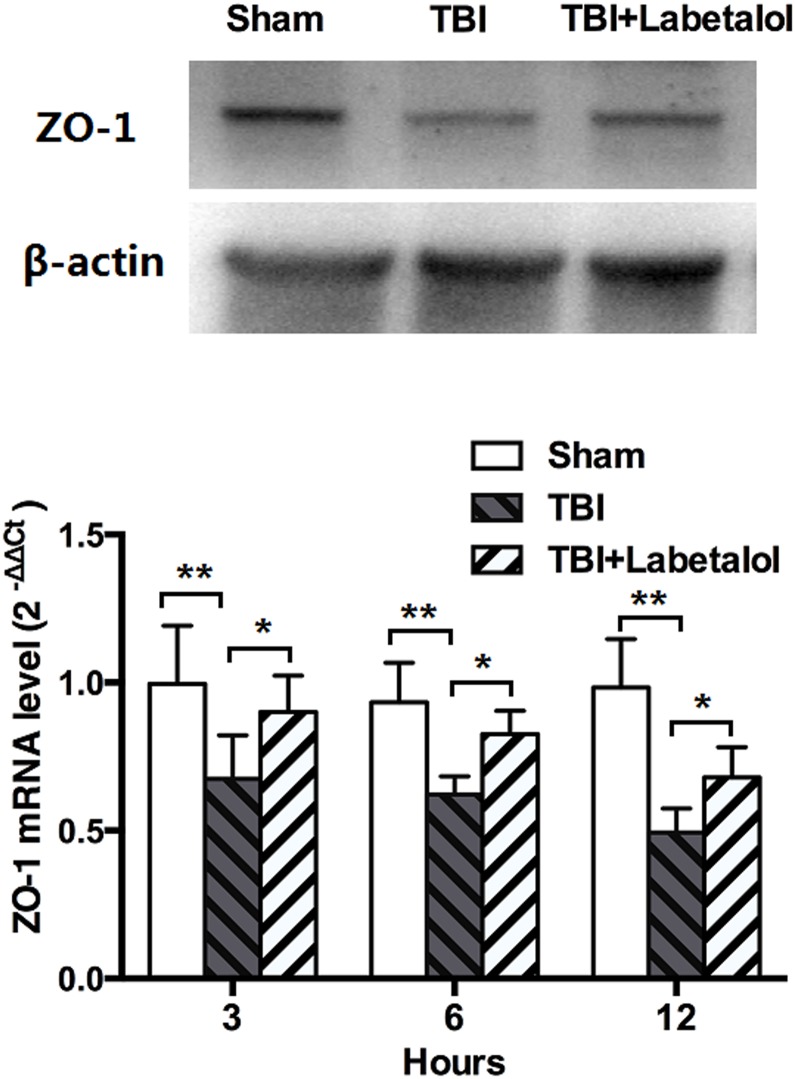
Intestinal ZO-1 expression in sham, TBI and TBI+labetalol groups at 3, 6 and 12h. ZO-1 protein (top panel, measured at 12h) and mRNA (bottom panel, measured at 3, 6 and 12h) expression in sham, TBI and TBI+labetalol groups. At all time-points, expression of ZO-1 was lower in rat distal ileumc of TBI vs. sham group, and labetalol significantly improved ZO-1 expression at all time-points (**p<0.001, *p<0.05).

As shown in Fig 4, expression of ZO-1 was lower in rat distal ileumc of TBI vs. sham group at all time-points. Labetalol significantly improved ZO-1 expression at all time-points, with 1.6-fold, 1.3-fold, and 1.4-fold higher in TBI with labetalol vs. TBI at 3h, 6h, and 12h, respectively (*p*<0.05).

### Histopathological Evaluation

**Fig 5:**
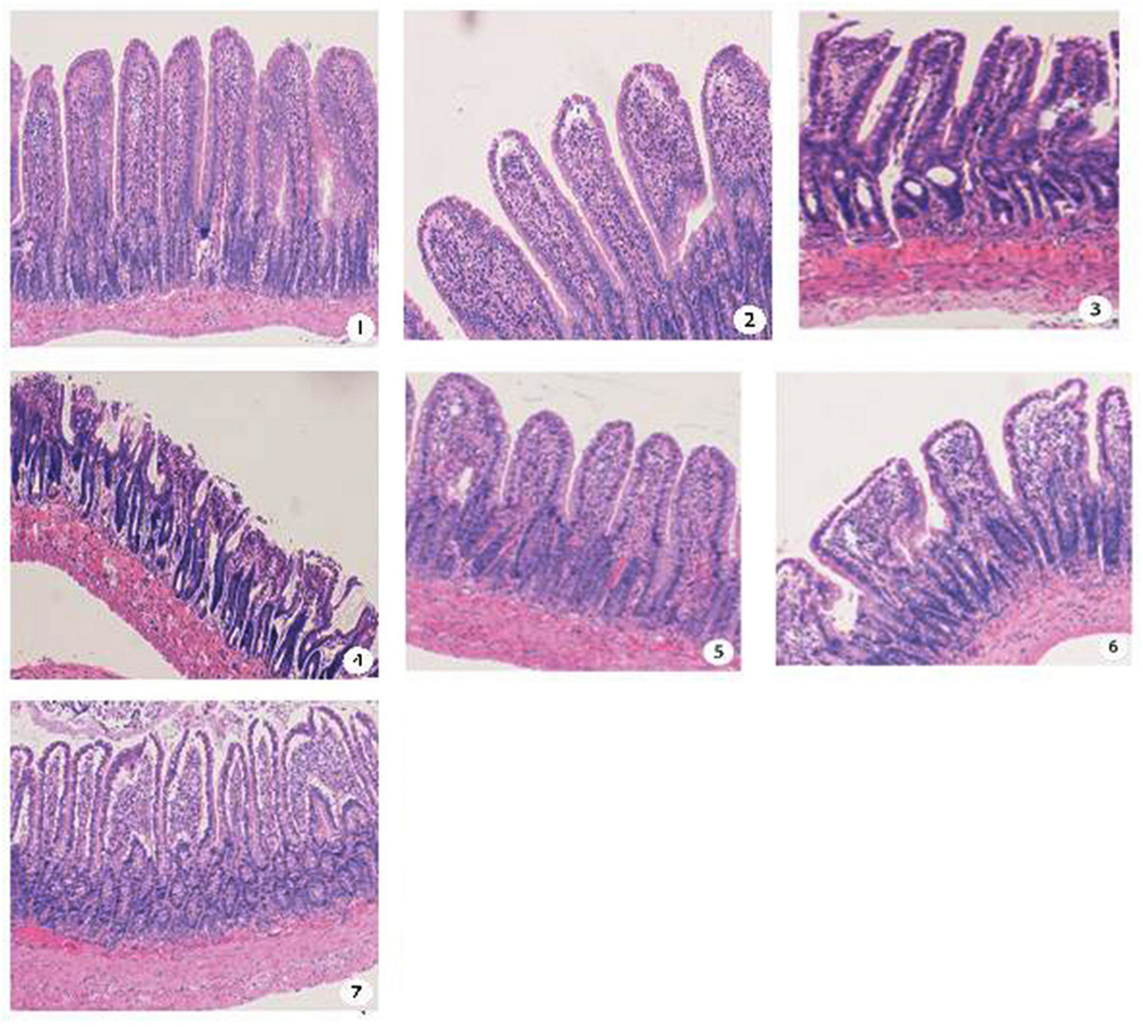
Terminal ileum H&E staining (all images are 50*magnification) at 3, 6, and 12h after either sham or TBI ±Labetalol. ① Terminal ileum in sham controls showing normal villi and consistent villous height;② At 3 h, intestinal mucosal villi with widened subepithelial space in TBI animals;③ At 6h, intestinal the top part of mucosal villi is damaged with central lacteal expansion in TBI animals; ④ At 12h, villous epithelium into a block off in TBI animals; ⑤ At 3 h, villous structure of the normal mucosa in TBI+labetalol animals; ⑥ At 6h, intestinal mucosal villi with widened subepithelial space in TBI+labetalol animals; ⑦ At 12h, villi with further expansion of the submucosal gap, villus tip epithelial elevation and damaged or broken top part in TBI+labetalol animals.

**Fig 6:**
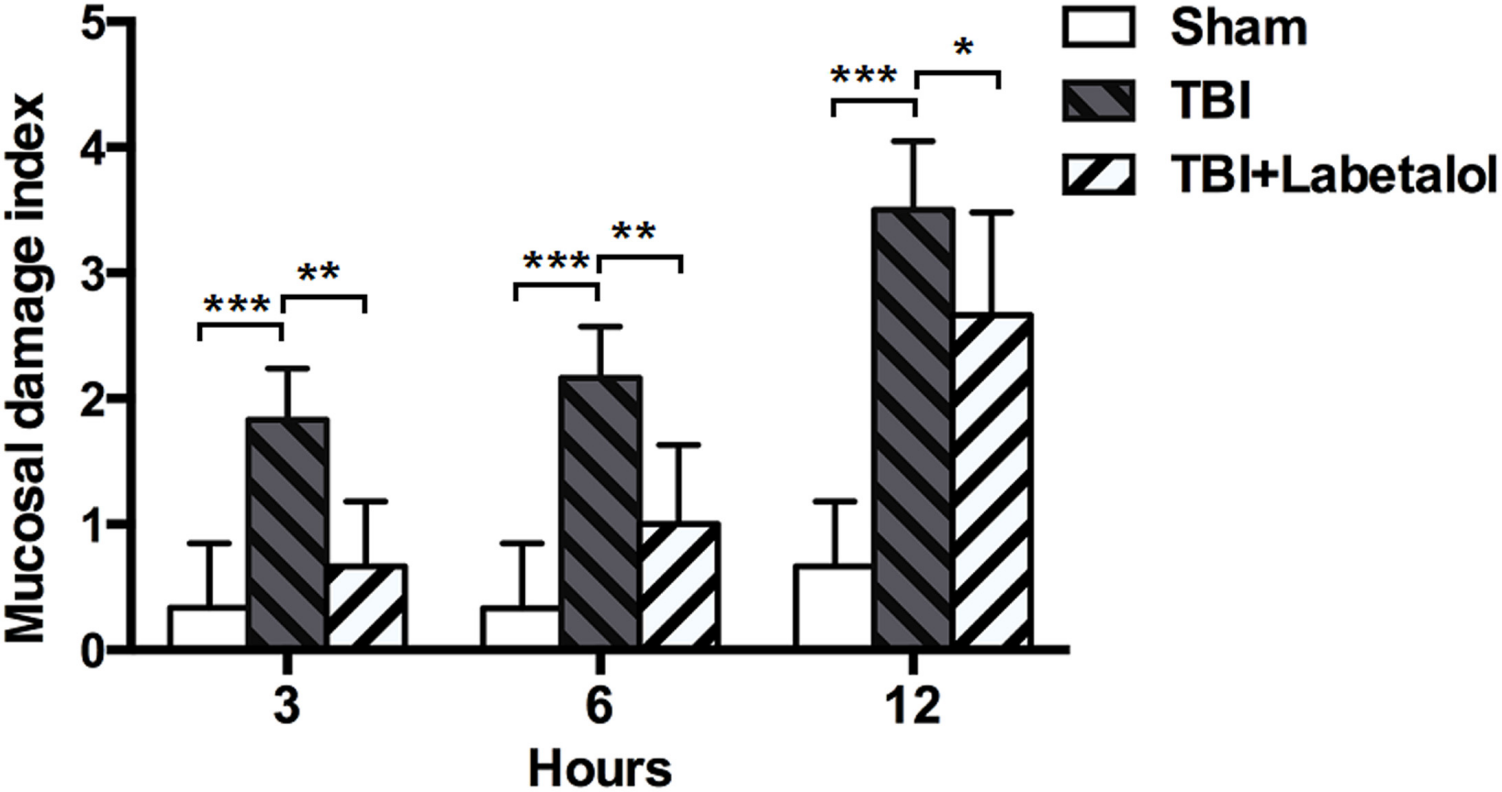
Mucosal damage index in sham, TBI and TBI+labetalol groups at 3, 6 and 12h. Mucosal damage index was determined according to Chiu's scoring criteria at 3, 6, and 12h after either TBI ±Labetalol or sham procedure. At each time-point, the mucosal damage index was higher in TBI groups (3h: 1.83±0.41; 6h: 2.17±0.41; and 12h: 3.50±0.55) when compared with sham groups (3h: 0.33±0.52; 6h: 0.33±0.52; and 12h: 0.66±0.52; ***p = 0.000). Labetalol intervention preserved intestinal architecture at each time-point. Mucosal damage index was lower in TBI with vs. without labetalol at 3 and 6h (0.67±0.52 vs. 1.83±0.41; and 1.00±0.63 vs. 2.17±0.41, respectively; **p<0.01), and at 12h (2.66±0.82 vs. 3.50±0.55; *p<0.05) after TBI.

The terminal ileum was harvested at 3, 6, and12h after either sham procedure or TBI ±Labetalol intervention for histological analysis using H&E staining (Fig 5). Normal appearing villi with normal villous height and no evidence of intestinal necrosis were observed in sham group at all time-points, while mucosal damage index was higher in TBI vs. sham group at all time-points (3h: 1.83±0.41 vs. 0.33±0.52; 6h: 2.17±0.41 vs. 0.33±0.52; and 12h: 3.50±0.55 vs. 0.66±0.52; *p* = 0.000). In contrast, labetalol intervention had a protective effects on intestinal architecture at all time-points with lower mucosal index damage in TBI with vs. without labetalol (3h: 0.67±0.52 vs. 1.83±0.41; 6h: 1.00±0.63 vs. 2.17±0.41; and 12h: 2.66±0.82vs. 3.50±0.55; *p*<0.01, *p* = 0.04) (Fig 6).

## Discussion

This study showed that labetalol has a protective effect against intestinal dysfunction after TBI. Treatment with labetalol reduced TBI-induced sympathetic hyperactivity, and prevented histopathological intestinal injury accompanied by changes in gut permeability and gut TNF-α expression in a rat model of TBI.

Recent literature has demonstrated a strong association between neurologic trauma and the development of non-neurologic organ dysfunction, which appears to be a result of sympathetic hyperactivity [28, 29], the surge in epinephrine tone and proinflammatory cytokines [8, 30]. Several investigators have evaluated the post-TBI state and noted a greater than sevenfold increase in norepinephrine, epinephrine, and their urine-excreted metabolites. Epinephrine level elevations appear to correlate with significant increases in sympathetic hyperactivity and are most pronounced during the first week after injury [31, 32]. Decreasing adrenergic tone through beta-blockade has been hypothesized to improve outcome after TBI [8]. To this end, Morel et al. [33] demonstrated that the hyperdynamic state mediated by sympathetic overactivity after severe injury was successfully reduced through the utilization of adrenergic blockade, and animal models have shown that pretreatment with beta-blockade reverses catecholamine-induced immunosuppression [34, 35]. In the present study, we showed that labetalol reduced plasma epinephrine and TBI-induced intestinal injury in rats. The principal physiologic action of labetalol is to competitively block adrenergic stimulation of β-receptors within the myocardium (β1-receptors) and within bronchial and vascular smooth muscle (β2-receptors), and α1-receptors within vascular smooth muscle. The rate limiting step in the synthesis of cathecolamines is subject to negative feedback by the end products, in our study by epinephrine. However, the exact mechanism that labetalol reduces plasma epinephrine level remains to be explored.

An intact intestinal epithelium is required to maintain an effective barrier against luminal bacteria that normally inhabit the gut and against endotoxin. The maintenance of intestinal barrier function is highly dependent on epithelial cell-to-cell adhesion, which is indispensable for intestinal architecture [36]. Tight junctions are located at the most apical part of the lateral membranes of epithelial and endothelial cells and comprise various molecules, such as the transmembrane proteins occludin [37], claudins [38], tricellulin [39] and the peripheral membrane proteins zonula occludens (ZOs). ZOs include three isoforms: ZO-1, ZO-2, and ZO-3 [40–43]. Zonula occludens protein 1 is a 220-kd tight junction protein that links the transmembrane protein occludin to the actin cytoskeleton within the apical portion of the cell and is a particularly important molecule in the formation of tight junctions [44, 45] and likely in the function of intestinal ones. Immunohistochemical alteration of ZO-1 is closely associated with increased intestinal permeability in patients with nonalcoholic fatty liver diseases [46]. Splanchnic hypoperfusion is a common phenomenon after trauma-induced stress, and surge in epinephrine tone is associated with gastrointestinal vasoconstriction, cramps, and significantly reduced intestinal mucosal blood flow,resulting gastrointestinal impairment including loss of intestinal tight junction proteins and increased gut permeability [1, 2]. Bansal et al. [1] showed that TBI-induced sympathetic hyperactivity and splanchnic hypoperfusion caused alteration of the intestinal tight junction proteins ZO-1 and occludin, which correlates to increased intestinal permeability and distinct changes. Moreover, TNF-α and other inflammatory factors is known to cause downregulation of ZO-1 [47] and induce intestinal mucosal injury by destroying the tight junctions between cells [48]. Costantini et al. [47] showed that pentoxifyline, a known anti-inflammatory agent, significantly decreased TNF-α levels and prevented an increase in intestinal permeability in a severe burn model. In our study, decreasing hyperactivity of adrenergic tone with labetalol decreased intestinal TNF-α level, and prevented an increase in intestinal permeability in the TBI rat model. In addition, myosin light chain kinase (MLCK) that plays an important role in junction protein function was also reported to be modulated by adrenergic blockers [14, 49]. Further research is needed to understand whether labetalol affects barrier function through modulation of MLCK.

It is important to note that our study is a hypothetical construct with inherent limitations. Animal grouping in our study was based on the published literatures [13, 14]. It is more reasonable to add a group that receives labetalol alone (no TBI) as an important control. Thus, this kind of animal grouping may have measurement bias. Another limitation was Labetalol dose that we used in this study. Only dose of 30mg/kg was chosen [15] and a dose-response wasn’t performed. This is relevant due to some of the minimal protective effects seen with the drug in some assays. Further research is needed to understand whether there is more protection at a higher dose. Furthermore, since changes in gut permeability and TNF-a in the TBI+Labetalol group were statistically significant compared to TBI group, the results of our study would be only applicable based on the small sample size. Additional studies based on large sample are needed to confirm the results. In addition, chloral hydrate as an anesthetic agent by single injection at the dose range used is shown early to alter gut functions in rats. Thus, we used an improved method of chloral hydrate anesthesia in rat by intermittent intraperitoneal injection, and did not found the influence on the parameters measured in our study.

In summary, our preliminary results in a rat model showed that labetalol decreased the plasma epinephrine levels and histopathological intestinal injury induced by TBI. Labetalol therefore appears to have potent a protective effect on intestinal function after TBI by reducing sympathetic hyperactivity.
